# A Rare Case Report of Herbal Medication Induced Pancreatitis

**DOI:** 10.7759/cureus.13558

**Published:** 2021-02-25

**Authors:** Ahmed Mowafy, Islam Younes, Ahmed Omran, Sherif Elkattawy, Ruhin Yuridullah

**Affiliations:** 1 Internal Medicine, Rutgers - New Jersey Medical School/Trinitas Regional Medical Center, Elizabeth, USA; 2 Clinical Research, University of Louisville, Louisville, USA; 3 Internal Medicine, St. Joseph's Univeristy Medical Center, Paterson, USA

**Keywords:** pancreatitis, herbal medication

## Abstract

Acute pancreatitis is an acute inflammation of the pancreas that varies in clinical manifestation from mild to life-threatening that may require hospitalization. A 56-year-old male patient with a past medical history of diabetes mellitus and osteoarthritis developed acute pancreatitis likely secondary to the use of herbal medication intended for weight loss. Other causes of pancreatitis were excluded. This report describes a case of herbal medication-associated pancreatitis after the exclusion of other causes. The incidence of herbal medication-associated pancreatitis is indeterminate due to inadequate literature on similar cases. The aim of this review is to describe the effect of herbal-based medicines and their counteraction on developing acute pancreatitis.

## Introduction

Acute pancreatitis is a common inflammatory condition, and one of the leading gastrointestinal causes of hospitalization in the United States [1]. The most common identified causes of pancreatitis are gallstones and alcohol abuse. But there are a few other less common conditions associated with acute pancreatic inflammation. In this report, we attempt to present one of the lesser-known causes of pancreatitis, e.g., herbal medications. Medication-associated pancreatitis is a rare, but documented, cause of acute pancreatitis [2,3]. In the literature review, most of the documented cases are involving pharmaceutical preparations, but there are a few cases of pancreatitis reported where no other cause can be identified, but the use of herbal medicines. However, before these incidences can be categorized as idiopathic inflammation of the pancreas, the involvement and role of these herbal medicines in the inflammatory process should be clarified.

## Case presentation

**Figure 1:**
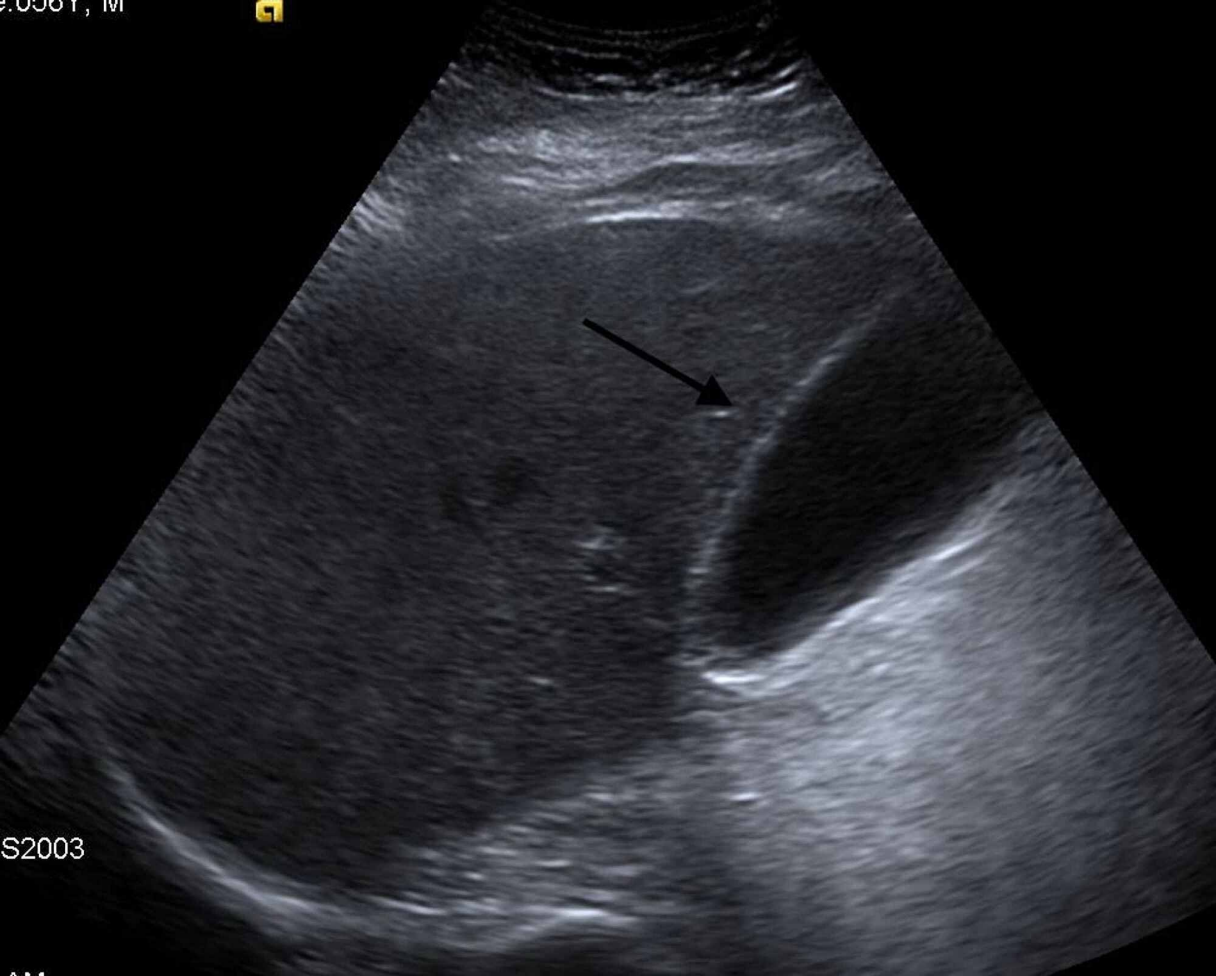
Gallbladder ultrasound shows no gallstones

**Figure 2:**
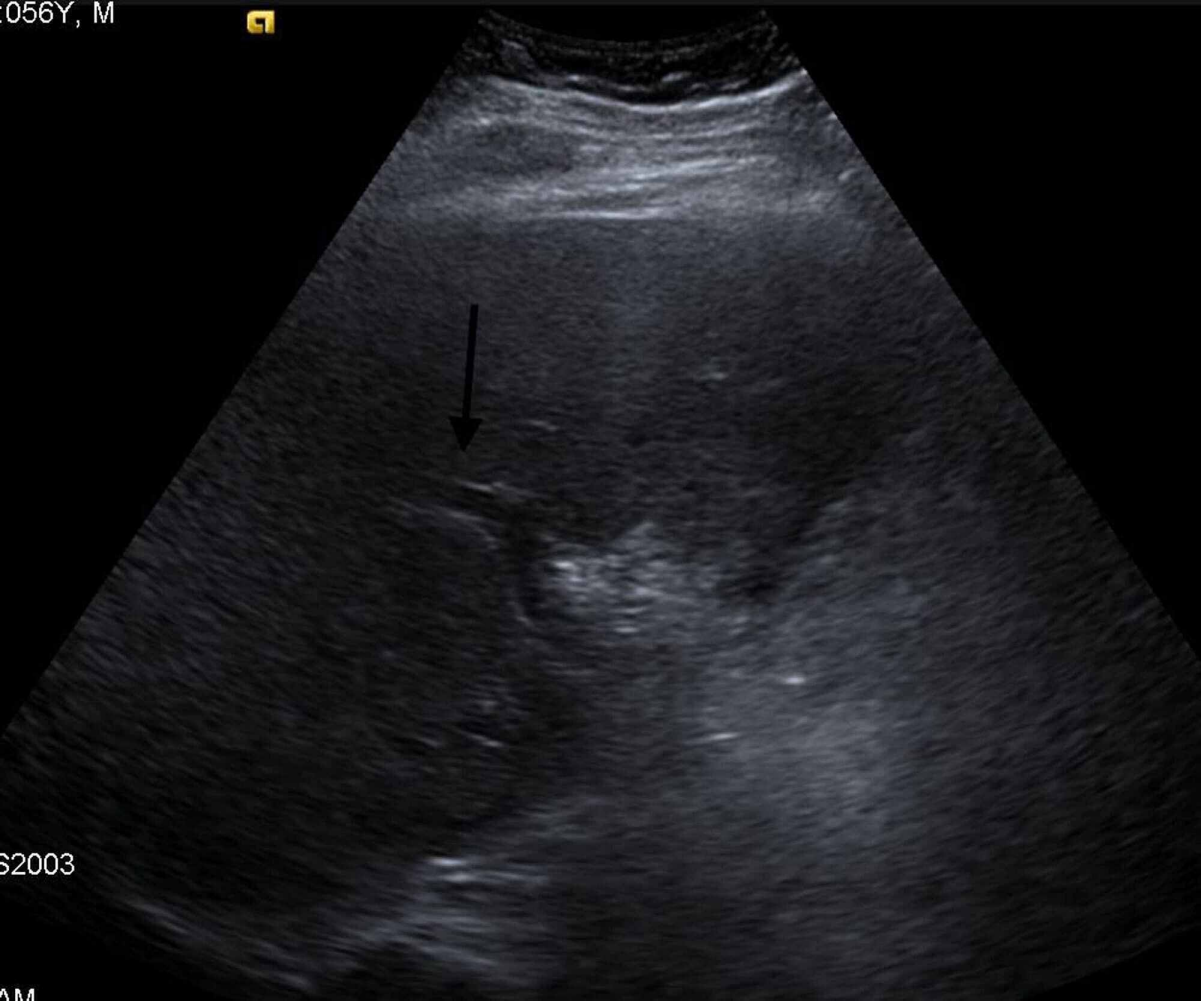
Gallbladder Ultrasound shows no common bile duct dilation

A 56-year-old male with a past medical history of diabetes mellitus and bilateral knee osteoarthritis, presented to the emergency department with severe abdominal pain for five hours, radiating to the back, associated with nausea and five episodes of non-bloody, non-bilious vomiting. He otherwise denied any other significant symptoms. He reported tobacco smoking of 25 pack-years, social drinking 1-2 beers on weekends, and denied any illicit drug use. The patient also denied any significant family history. On presentation, he was afebrile, hemodynamically stable, awake, alert, and oriented; however, on physical examination, he had abdominal distension and generalized tenderness. Lab work was remarkable for leukocytosis, with white blood cells (WBC) 14.7 k/ul (normal range: 4.8-10.8 K/UL), hemoglobin (Hb) 16.5 gm/dl (normal range: 12-17 gm/dl) with haematocrit (Hct) 49.8 % (normal range: 37-51%), creatinine 1.6 mg/dl (normal range: 0.7-1.2 mg/dl), blood urea nitrogen (BUN) 27 mg/dl (normal range: 8-20 mg/dl) and lipase 4200 U/L (normal range 0-160 U/L). The patient was admitted to the medical floors for IV fluids and analgesic therapy for acute pancreatitis management. Further studies were done, showing unremarkable lipid panel with normal liver function tests. As seen in Figure 1 & 2, gallbladder ultrasound showed no gallstones or common bile duct (CBD) dilatation, respectively. 

**Figure 3:**
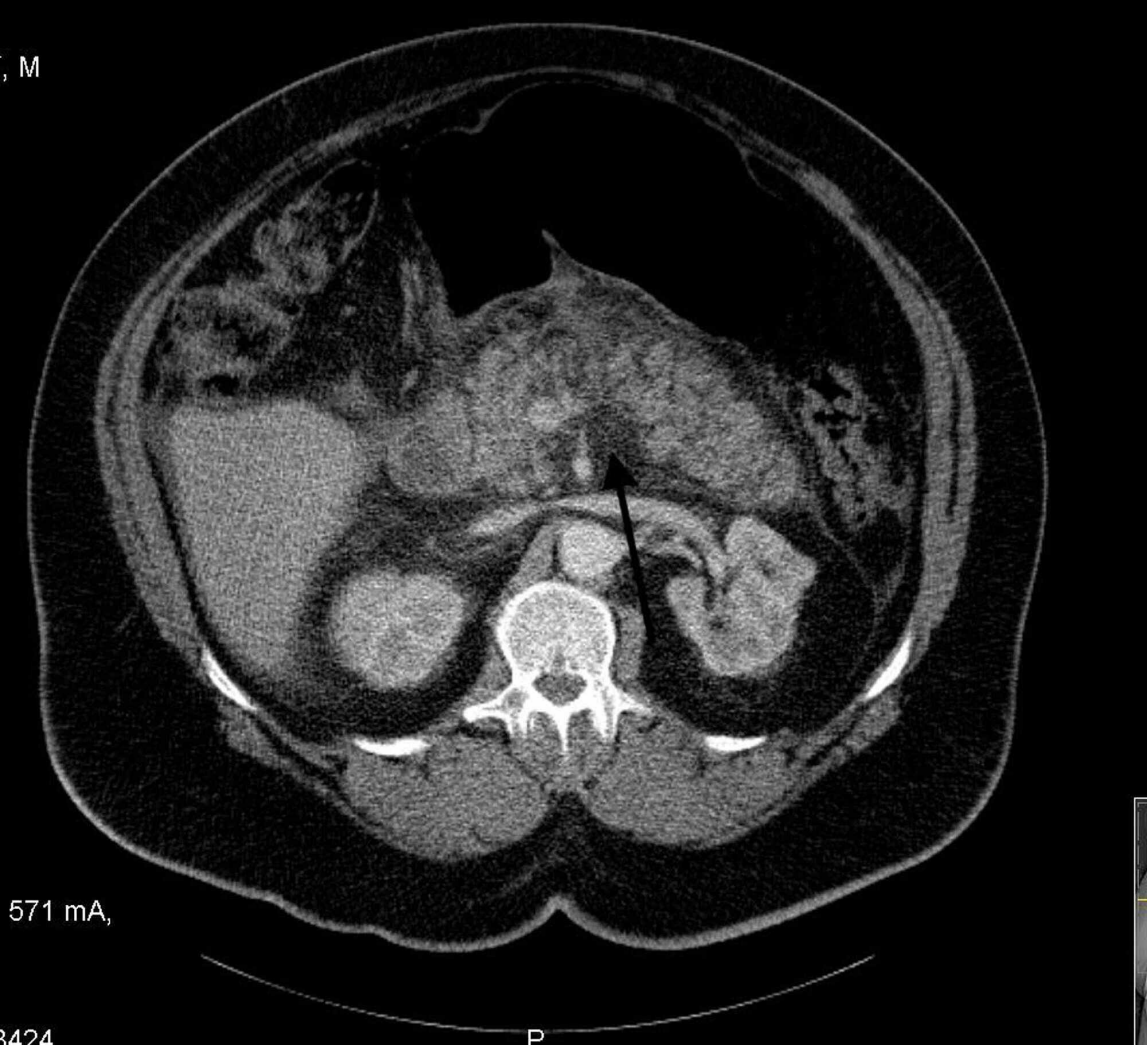
CT abdomen shows peripancreatic inflammation with focal fluid collection

CT abdomen showed acute peripancreatic inflammatory changes as seen in Figure 3, supporting the diagnosis of acute pancreatitis.

Upon further interrogation, the patient reported recent daily use of different kinds of herbs including fenugreek, turmeric, and Gymnema, to decrease his weight and blood sugar. Throughout the hospital stay, the patient's clinical status improved and he tolerated oral intake. He was recommended to stop taking the aforementioned herbs and discharged with instructions to follow up outpatient. 

## Discussion

Herbal medications' effect on glucose metabolism and their anti-diabetic properties have been studied sparsely [4], among these medications are the ones based on Gymnema plant extract [5]. Other studies focused more on the effects of certain plants, namely fenugreek & turmeric on the pancreas [6,7]. Medicines generated from these plants interfere with endocrine pancreatic structure and functions, with the ultimate goal of boosting the intrinsic mechanisms of glucose metabolism. Our patient had been using metformin for non-insulin dependent diabetes mellitus control and had resorted to herbal medicines for weight loss and lowering blood sugar. Given the studied effects of the three medicines the patient had used on pancreatic function, it would not be farfetched to assume these medicines would possibly have triggered an inflammatory cascade within the pancreas. Fenugreek was postulated to have a role in increasing pancreatic insulin secretion [8], an effect that, on a prolonged course, could possibly stress the pancreas to the point of inflammation. Turmeric has been studied to have an anti-inflammatory effect through blocking the activation of certain cytokines, namely Tumor Necrosis Factor Receptor 1 (TRAF1)/Apoptosis signal-regulating kinase 1 (ASK1)/c-Jun N-terminal kinase (JNK)/Nuclear factor kappa-light-chain-enhancer of activated B cells (NF-κB), signaling pathway [7]. Gymnema is also proved to have a protective effect on the pancreatic beta cells from apoptosis and other manors of cell death [9]. This level of interference and manipulation of inflammatory mediator activity could possibly lead to immune dysregulation, ultimately resulting in autoimmunity and pancreatic inflammation. Unfortunately, the biochemical and immunological mechanisms by which these medications execute their effects are severely understudied, and further inspection and understanding of these mechanisms are duly warranted.

## Conclusions

Botanic-based medicines are unregulated, and their wide spectrum of effects is largely unstudied. However, they remain somewhat popular in some cultures and used commonly enough to cause confusion when it comes to accurately identify a cause of some common illnesses like acute pancreatitis. Here, we demonstrate an average person who developed acute pancreatitis without classically fitting into one of the established categories of risk factors or triggers. In light of this report, we cannot emphasize enough the importance of further investigation of these herbal medicines and their adverse effects on the general population. More effort and research should be applied to identify and report similar cases, to raise awareness, and grow knowledge about herbal medicine-associated pancreatitis.
